# Association of *CTLA-4* (AT)n Variants in Basal Cell Carcinoma and Squamous Cell Carcinoma Patients from Western Mexico

**DOI:** 10.3390/cimb46080493

**Published:** 2024-08-01

**Authors:** Jose Manuel Rojas-Diaz, Marianela Zambrano-Román, Jorge Ramón Padilla-Gutiérrez, Yeminia Valle, José Francisco Muñoz-Valle, Emmanuel Valdés-Alvarado

**Affiliations:** 1 Instituto de Investigación en Ciencias Biomédicas, Centro Universitario de Ciencias de la Salud, Universidad de Guadalajara, Guadalajara 44340, Jalisco, Mexicodrjosefranciscomv@cucs.udg.mx (J.F.M.-V.); 2 Instituto de Investigación en Enfermedades Crónico Degenerativas, Departamento de Biología Molecular y Genómica, Centro Universitario de Ciencias de la Salud, Universidad de Guadalajara, Guadalajara 44340, Jalisco, Mexico

**Keywords:** basal cell carcinoma, squamous cell carcinoma, genetic variants, *CTLA-4*

## Abstract

The incidence of basal cell carcinoma (BCC) and squamous cell carcinoma (SCC) is constantly increasing, becoming a significant health problem. CTLA-4 is a critical immune checkpoint, and it has been suggested that a variant of variable-number tandem repeat in the 3’-UTR of its gene, known as (AT)n, may be associated with a higher susceptibility to some cancers; however, little is known about genetic variants of the *CTLA-4* gene in NMSC. To establish the association of this genetic variant in the *CTLA-4* gene with the susceptibility of NMSC carcinogenesis in the Western Mexican population, samples from 150 BCC patients, 150 SCC patients, and 150 healthy individuals as the reference group (RG) were analyzed by endpoint PCR, followed by electrophoresis to genotype the samples. We found that the short-repeat 104/104 bp genotype may be a risk factor for BBC carcinogens (OR = 2.92, *p* = 0.03), whereas the long-repeat 106/106 bp genotype may be a protective factor for both BCC (OR = 0.13, *p* = 0.01) and SCC (OR = 0.32, *p* = 0.01) susceptibility. Our results show that in the Western Mexican population, long-repeat (AT)n variants in the *CTLA-4* gene are associated with a protective factor in BCC and SCC. In contrast, short repeats are associated with a risk factor.

## 1. Introduction

Nonmelanoma skin cancers (NMSC) are the most common type of malign neoplasms worldwide, and their incidence is constantly increasing, being now a major public health problem [1]. The two most common forms of NMSC arise from the malignant transformation of basal and squamous cells, which are, respectively, termed basal cell carcinoma (BCC) and squamous cell carcinoma (SCC). Approximately 80% of skin cancers are BCC, the most diagnosed dermatological neoplasms, whereas approximately 15–20% are SCC [2]. In Mexico, BCC is the most common dermatological cancer (74%), followed by SCC (14%). Notwithstanding the poor register practice in Mexico in NMSC, the incidence of cases seems to be higher yearly [3].

Regardless of whether NMSCs are multifactorial diseases, some factors may increase the risk of carcinogenesis. UV radiation exposure is considered the major risk factor for developing skin cancers [4]. However, some genetic conditions and related signaling pathway alterations also contribute to BCC and SCC development [5]. Several variants in the genes that codify immune proteins are highly related to NMSC. Our work team has found associations between some genetic variants in the gene that codes for the MIF cytokine with the development of BCC [6] and SCC [7]; however, some genetic variants in other immune proteins may have a critical effect on NMSC.

CTLA-4 is a crucial regulator of immune response, acting as an inhibitory receptor on T-cells. It modulates immune activity by interacting with its ligands, CD80 and CD86, on antigen-presenting cells. CTLA-4 primarily functions by competing with CD28, mediating the trans-endocytosis of CD80 and CD86, and inducing tolerogenic effects that inhibit T-cell costimulation [8]. Research has shown that persistent CTLA-4 expression in neoplastic cells from cancer patients can contribute to disease progression [9].

In 1996, Allison and colleagues reported that CTLA-4 blockade had antitumor effects [10]. After a decade of clinical testing, Ipilimumab, an anti-CTLA-4 antibody, was proven to improve melanoma patient survival in a large Phase III clinical trial [11] and became the first checkpoint inhibitor approved by the FDA for advanced melanoma [12]. This breakthrough established CTLA-4 as a significant target for cancer therapies.

The *CTLA-4* gene is located at 2q33 and contains several genetic variants that are known to influence its genetic expression and protein activity [13]. Some genetic variants of this gene can contribute to the development of autoimmune disorders [14] and cancers [15].

Despite the *CTLA-4* gene having more than 100 variants, in recent years, a genetic variant of variable-number tandem repeat (VNTR) located in the 3′-untranslated region (3’-UTR) of exon 4, known as (AT)n, has been found to have an essential role in genetic expression and be associated with a higher susceptibility to some types of cancer. Despite little being known about genetic variants of the *CTLA-4* gene in NMSC, it has been established that long-repeat alleles in the (AT)n variants of the *CTLA-4* gene have significantly less mRNA expression that could lead to lymphoproliferative disorders that may end up in immune diseases [16], while short-repeat alleles in this gene are associated with higher mRNA stability thus contributing to impaired T-cell activation that may lead to a higher susceptibility to some types of cancer [17].

Although a few investigations link (AT)n variants to some types of cancer, the correlation between NMSC and these genetic variants in a Mexican population has never been found. Therefore, this study aims to establish the association between the presence of the (AT)n variants in the *CTLA-4* gene and the susceptibility of BCC and SCC carcinogenesis in people from Western Mexico.

## 2. Materials and Methods

### 2.1. Patients

A total of 450 individuals from Western Mexico, spanning three generations including their own, participated in this research project: 150 patients with histologically confirmed basal cell carcinoma (BCC), 150 patients with squamous cell carcinoma (SCC), both unrelated, as well as 150 age- and sex-matched individuals from the same area without cancer or skin diseases, serving as the reference group (RG). The whole study was conducted in accordance with the Declaration of Helsinki, and all participants provided informed consent by signing voluntarily.

### 2.2. Genomic DNA

Genomic DNA samples were extracted from leukocytes by Miller’s modified technique using peripherical blood obtained by venipuncture [18]. Endpoint PCRs were performed to analyze the (AT)n variants of the *CTLA-4* gene. The optimal PCR conditions consisted of an initial 95 °C denaturation for 5 min followed by 30 cycles of denaturation at 95 °C for 30 s, annealing at 65 °C for 30 s, and elongation at 72 °C for 30 s, with a final elongation at 72 °C for 7 min. The primers used (Invitrogen^®^, Carlsbad, CA, USA) were: 5′ GCC AGT GAT GCT AAA GGT TG 3′ (forward) and 5′ AAC ATA CGT GGC TCT ATG CA 3′ (reverse) [19]. Electrophoreses on 7% polyacrylamide gels were run with the amplification products, and they were subsequently genotyped.

### 2.3. Statistics

The Hardy–Weinberg equilibrium test was performed, and genotype and allele frequencies were calculated by the χ^2^ or Fisher’s exact test, when applicable. Odds ratios (OR) and 95% confidence intervals (95% CI) were calculated to test the probability that the genotype and allele frequencies were associated with BCC and SCC. A *p*-value < 0.05 was considered to be statistically significant. All the statistical analyses were done with the PASW v18.0 statistical program (SPSS Inc., Chicago, IL, USA).

## 3. Results

All clinical characteristics and demographic data are shown in Table 1. The median age of BCC and SCC groups was 67 (28–96) and 71 (38–96) years old, respectively. The sex distribution in BCC patients was 63% female and 37% male, while 45% female and 55% male in SCC patients. In the case of BCC, most of the tumor sizes were considered high-risk (67%), while in SCC, the proportion between high- and low-risk tumor sizes was practically the same. In both NMSCs, most tumors were localized in body parts continuously exposed to UV radiation, such as the head and neck (93% in BCC and 64% in SCC), followed by core and upper extremities (5% in BCC and 25% in SCC). In both cancers, a histopathologic test showed that most of the tumors had a low risk (66% in BCC) or were highly differentiated (61% in SCC).

The χ^2^ analysis was used to compare the observed number of genotypes with that expected for a population in a Hardy–Weinberg equilibrium. The χ^2^ analysis was also employed to test the significance of the differences in the observed alleles and genotypes between groups. No deviation from the Hardy–Weinberg equilibrium was detected in the reference group (*p* > 0.05). The Mann–Whitney U test was used to compare the mean values.

A total of 14 different genotypes were found in BCC genotypification, and the 88/88 bp genotype was the most concurrent in patients (74%) as well as in the reference group (77.3%). The statistical analysis showed that in BCC, the 104/104 bp variant (short repeat) may be a risk factor (OR = 2.92, *p* = 0.03), whereas the 106/106 bp variant (long repeat) may be a protective factor (OR = 0.13, *p* = 0.01). Similar data were found in allelic frequencies where the 104 bp allele may be a risk factor in the BCC developing (OR = 2.57, *p* = 0.01) while the 106 pb allele may be a protective factor (OR = 0.11, *p* = 0.00). The allele and genotype frequencies in BCC patients and the reference group are shown in Table 2.

In the case of SCC, 16 different genotypes were found after genotypification. Also, the 88/88 bp genotype was the most common in patients (73.3%) as well as in the reference group (78%). The statistical analysis showed that in SCC, the 106/106 bp variant (long repeat) may also be a protective factor (OR = 0.32, *p* = 0.01). The 106 bp allele may be a protective factor as well (OR = 0.33, *p* = 0.00). The allele and genotype frequencies in SCC patients and the reference group are shown in Table 3.

## 4. Discussion

Our results show that in Western Mexico, the incidence of BCC is higher in women, which is consistent with the data found by Hernández-Zárate et al. [3]. By contrast, the incidence of SCC is higher in men, as other authors have reported [20]. Some studies have linked the NMSC to sex due to the higher incidence in men. However, lifestyle highly contributes to the development of NMSC, considering UV exposure as the primary etiological factor, and in some countries like Mexico, outdoor activities have been historically undertaken by men [21]. It is also essential to highlight the new tanned-skin trend that seems to be increasing mainly among women, as well as the use of tanning beds, which may contribute to skin neoplasm development [22]. Most of the neoplasms were found in highly sun-exposed body zones such as the head, neck, core, and upper extremities (98% in BCC and 89% in SCC). This pattern is constantly viewed in skin cancers, and it is evident that UV ray exposure plays a major etiological factor [4]. We found a median age of 67 years old in BCC and 71 in SCC patients at the moment of diagnosis. As other authors have mentioned, the development of these neoplasms in older ages may be due to the accumulation of the damage induced by UV rays over the years [23].

Some studies have established CTLA-4 as a critical molecule by regulating the development of UV-induced tolerance and, concomitantly, skin cancers [15], but its importance goes further to other cancers. Other authors have found a strong linkage between *CTLA-4* genetic variants and some malignant tumors [9], like osteosarcoma [24], gastric and colorectal cancers [25,26], cervical squamous cell carcinoma [17], and thyroid cancer [27]. However, the role of the (AT)n variant in the *CTLA-4* gene remains controversial. This genetic variant is highly polymorphic, and it may vary depending on the studied population. We found 9 alleles in BCC and 10 in SCC, but authors like Barreto et al. have found 21 alleles in the Spanish population [28], while Machida et al. reported 7 alleles in the Asian population [19], and Al Fadhli and collaborators found only 2 alleles in the Middle East population [29].

Our analysis suggests that in BCC, the 104/104 variant (short repeat) confers a risk to BCC development, while the 106/106 (long repeat) seems to be a protective factor. In SCC, we also found that the 106/106 variant seems to be a protective factor in carcinogenesis. It has been established that the size of the dinucleotide (AT) repeats in the 3’-UTR affects mRNA stability and, subsequently, the rate of translation [30]. Some authors have reported that short-repeat (AT)n variants in the *CTLA-4* gene are associated with a higher mRNA stabilization that contributes to lower activation of T-cells, which may result in an impaired antitumor response [17].

In the same way, longer-repeat (AT)n variants may be associated with a lower mRNA stabilization that could reduce the expression of CTLA-4 [16] and ultimately allow the activation of T-cells, improving antitumoral response. Jong et al. [31] have transduced T-cells with long (AT)n elements and demonstrated that the long-repeat (AT)n variant leads to a reduction of mRNA, with phenotypic consequences in T-cell activation. Similarly, Malquori et al., using luciferase reporter assays, found that (AT)n variants in the *CTLA-4* gene can influence mRNA stability and translation efficiency by post-transcriptional mechanisms that may provide cells with tools to rapidly respond to changes in intracellular and extracellular stimuli [32].

Despite the role of the different genetic variants in the *CTLA-4* gene having not totally been elucidated, it is well established that polymorphisms in the *CTLA-4* gene confer susceptibility to cancers. It has been postulated that some of these polymorphisms may influence rates of endocytosis, surface trafficking, glycosylation, and intracellular/surface partitioning [33].

It has been proposed that some polymorphisms in the *CTLA-4* gene could be considered to be biomarkers of cancer susceptibility [34]; however, it has also been proposed that some genetic variants in this gene may serve as potential biomarkers predictive of favorable outcomes in melanoma patients treated with ipilimumab [35]. This finding is specifically relevant considering the recent approval by the FDA of immune checkpoint blockade immunotherapies for both BCC and SCC [36]. Immunotherapy is playing an increasingly critical role in the management of advanced disease and is considered the standard of care for upfront systemic therapy in locally advanced and unresectable NMSC [37].

While some work groups have found an association between some *CTLA-4* variants and cancer, like the single nucleotide polymorphisms (SNP) +49 A/G in exon 1, −1661 A/G, −318 C/T, and −17,722 T/C in the promoter region, or CT60A/G in the 3′-UTR [38], we are showing for the very first time the association between the VNTR (AT)n in NMSC. These findings enhance our understanding of how *CTLA-4* variants contribute to NMSC carcinogenesis, suggesting potential implications for therapy and prognosis. However, our study is limited by the lack of mRNA expression evaluation and protein quantification. Future research should address these gaps to fully elucidate the role of *CTLA-4* variants in the development and progression of basal cell carcinoma (BCC) and squamous cell carcinoma (SCC). Further functional analyses are necessary to uncover the molecular mechanisms underlying the influence of the (AT)n variant in NMSC.

## 5. Conclusions

Our study reveals that short repeats of the (AT)n variant in the 3’-UTR of the *CTLA-4* gene are associated with an increase in the risk of nonmelanoma skin cancer (NMSC), while long repeats may provide a protective effect.

## Figures and Tables

**Table 1:** Demographics and clinical characteristics of BCC and SCC patients.

Variable	BCC (n = 150)	SCC (n = 150)
**Demographics**		
Age	67 (26–92)	71 (38–96)
Sex	Male: 37% (56) Female: 63% (94)	Male: 55% (83) Female: 45% (67)
**Clinical characteristics**		
**Tumor size**		
High risk	≥5 mm: 67% (100)	≥2 cm: 49% (74)
Low risk	≤5 mm 33% (50)	≤2 cm: 51% (76)
**Tumor localization**		
Head and neck	93% (140)	64% (96)
Core and upper extremities	5% (7)	25% (38)
Other body parts	2% (3)	11% (16)
**Histopathology**		
**BCC histopathology**		
Low risk	66% (99)	-
High risk	34% (51)	-
**SCC histopathology**		
Highly differentiated	-	61% (92)
Moderately differentiated	-	10% (15)
Lowly differentiated	-	1% (1)
Bowen’s disease	-	26% (39)
Other	-	2% (3)

**Table 2:** Genotype and allele frequencies of (AT)n variant of *CTLA-4* gene in BCC patients and the reference group.

Variant	% BCC (n = 150)	% RG (n = 150)	OR (CI 95%); *p*
***CTLA-4* (AT)n**			
**Genotype**			
88/88	74 (111)	77.3 (116)	1
88/102	2 (3)	0 (0)	7.31 (0.37–143); 0.07
88/104	2 (3)	0 (0)	7.31 (0.37–143); 0.07
88/108	2 (3)	0 (0)	7.31 (0.37–143); 0.07
88/122	1.3 (2)	0 (0)	5.22 (0.24–110.03); 0.15
96/96	0.7 (1)	0.7 (1)	1.04 (0.06–16.9); 0.9
102/108	1.3 (2)	0 (0)	5.22 (0.24–110.03); 0.15
102/122	1.3 (2)	0 (0)	5.22 (0.24–110.03); 0.15
**104/104**	**9.4 (14)**	**3.3 (5)**	**2.92 (1.02–8.39); 0.03**
104/106	0 (0)	1.3 (2)	0.20 (0.01–4.40); 0.16
**106/106**	**2 (3)**	**16 (24)**	**0.13 (0.03–0.44); 0.01**
108/108	2.7 (4)	0.7 (1)	4.18 (0.46–37.98); 0.16
108/110	0 (0)	0.7 (1)	0.35 (0.01–8.64); 0.32
108/112	1.3 (2)	0 (0)	5.22 (0.24–110.03); 0.15
**Allele**			
88	77.7 (233)	77.3 (232)	1
96	0.7 (2)	0.7 (2)	0.99 (0.13–7.12); 0.99
102	2.3 (7)	0 (0)	14.93 (0.84–263.01); 0.01
**104**	**10.3 (31)**	**4 (12)**	**2.57 (1.28–5.13); 0.01**
**106**	**2 (6)**	**16.7 (50)**	**0.11 (0.05–0.28); 0.00**
108	5 (15)	1 (3)	4.9 (1.42–17.42); 0.01
110	0 (0)	0.3 (1)	0.33 (0.01–8.1); 0.31
112	0.7 (2)	0 (0)	4.97 (0.23–104); 0.15
122	1.3 (4)	0 (0)	8.96 (0.48–167); 0.04

**Table 3:** Genotype and allele frequencies of (AT)n variant of *CTLA-4* gene in SCC patients and the reference group.

Variant	% SCC (n = 150)	% RG (n = 150)	OR (CI 95%); *p*
***CTLA-4* (AT)n**			
**Genotype**			
88/88	73.3 (110)	78 (117)	1
96/96	0 (0)	1.3 (2)	0.23 (0.01–4.28); 0.16
102/102	0 (0)	0.7 (1)	0.339 (0.014–8.41); 0.32
104/104	6 (9)	4 (6)	1.53 (0.52–4.42); 0.43
**106/106**	**4.6 (7)**	**14.6 (22)**	**0.32 (0.13–0.78); 0.01**
108/108	2 (3)	0 (0)	7.12 (0.36–139.4); 0.08
122/122	0.7 (1)	0 (0)	3.05 (0.12–75); 0.31
132/132	0.7 (1)	0 (0)	3.05 (0.12–75); 0.31
88/102	2 (3)	0 (0)	7.12 (0.36–139.4); 0.08
88/104	2 (3)	0 (0)	7.12 (0.36–139.4); 0.08
88/122	2 (3)	0 (0)	7.12 (0.36–139.4); 0.08
88/132	1.3 (2)	0 (0)	5.08 (0.24–107.11); 0.15
102/108	2 (3)	0 (0)	7.12 (0.36–139.4); 0.08
104/106	0.7 (1)	0.7 (1)	1.01 (0.06–16.46); 0.9
108/110	0.7 (1)	0.7 (1)	1.01 (0.06–16.46); 0.9
108/112	2 (3)	0 (0)	7.12 (0.36–139.4); 0.08
**Allele**			
88	77 (231)	78 (234)	1
96	0 (0)	1.3 (4)	0.113 (0.006–2.10) 0.05
102	2 (6)	0.7 (2)	3.03 (0.61–15.21) 0.15
104	7.4 (22)	4.4 (13)	1.71 (0.84–3.45) 0.13
**106**	**5 (15)**	**15 (45)**	**0.33 (0.18–0.62) 0.00**
108	4.3 (13)	0.3 (1)	13.16 (1.71–101.48) 0.01
110	0.3 (1)	0.3 (1)	1.01 (0.06–16.22) 0.99
112	1 (3)	0 (0)	7.09 (0.36–138) 0.08
122	1.7 (5)	0 (0)	11.14 (0.61–202) 0.03
132	1.3 (4)	0 (0)	9.11 (0.48–170) 0.05

## Data Availability

Data are available upon request.
